# Supplementary material for: Patterns of Intron Gain and Loss in Fungi
Source: PLoS Biol. 2004 Nov 30;2(12):e422. doi: 10.1371/journal.pbio.0020422 (PMC532390; doi:10.1371/journal.pbio.0020422)
Supplement: Table S1 — Also available at http://genes.mit.edu/NielsenEtAl/. (4.3 MB ZIP). [file pbio.0020422.st001.zip › NielsenEtAl/html/840.html]

AN0570.1.NCU03565.1.MG03965.1.FG10845.1


```
 CLUSTAL W (1.82) Multiple Sequence Alignments - Introns Inserted


Sequence 1: NCU03565.1	136 aa
Sequence 2: MG03965.1	175 aa
Sequence 3: FG10845.1	136 aa
Sequence 4: AN0570.1	134 aa
Alignment Length: 175 aa
Number Identitical Residues: 88 aa
Alignment Score (without introns) 3584


MG03965.1 	MVSRLYGRRSSLLSPHSFRRQPPSQILTPLSQTAAAVTMVKVTNKT1LHSSRRKSRKAHF
NCU03565.1	--------------------------------------MVKVNN-S1VSSSRRKARKAHF
FG10845.1 	--------------------------------------MTKINP-S1VASSRRKSRAAHF
AN0570.1  	---------------------------------------MAVTNQA1ISSSRRKSRKAHF
          	                                         :. .: : *****:* ***

MG03965.1 	GAPSSVRRNIMSAPLSKELREKYN0VRSIPIRKDDEVQIVRGSNKDKEGKVTSVYRLKYV
NCU03565.1	SAPSSVRRNIMSAPLSKELREKYN0VRSIPIRKDDEVTIVRGSNKDKEGKVTSVYRLKYV
FG10845.1 	KAPSDQRRVIMSAPLSKELREQYN0VRSIPIRKDDEVTIVRGSNKGREGKITSVYRLKYV
AN0570.1  	NAPSSVRRVIMSAPLSKELREKHN0VRSIPIRKDDEVTIVRGTNKGREGKITSVYRLKWC
          	 ***. ** ************::* ************ ****:**.:***:*******: 

MG03965.1 	IHVERVTREKTTGASVPLGIHPSNVVITKLKLDKDREDILARIKAGRVTSGKAKSSE
NCU03565.1	IHVERVTRDKATGASVPVGIHPSNVVITKLKLDKDREAILERIKRGRELAKPTKVSA
FG10845.1 	VHVERVTRDKANGQSVPLGIHPSNCVITKLKLDKDREDILRRSKVGRELAANNKVTA
AN0570.1  	VHVERVVREKSNGQSVPLPIHPSKVVITKLKLDKDREQILERISKGRE-AVKAKSA-
          	:*****.*:*:.* ***: ****: ************ ** * . **  :   * :
```
